# Genomic characterization of a new phage BUCT541 against *Klebsiella pneumoniae* K1-ST23 and efficacy assessment in mouse and *Galleria mellonella* larvae

**DOI:** 10.3389/fmicb.2022.950737

**Published:** 2022-09-16

**Authors:** Mingfang Pu, Yahao Li, Pengjun Han, Wei Lin, Ronghua Geng, Fen Qu, Xiaoping An, Lihua Song, Yigang Tong, Shuyan Zhang, Zhen Cai, Huahao Fan

**Affiliations:** ^1^College of Life Science and Technology, Beijing University of Chemical Technology, Beijing, China; ^2^Beijing Advanced Innovation Center for Soft Matter Science and Engineering (BAIC-SM), Beijing University of Chemical Technology, Beijing, China; ^3^Aviation General Hospital, Beijing, China; ^4^Department of Medical Technology Support, Jingdong Medical District of Chinese People's Liberation Army of China General Hospital, Beijing, China

**Keywords:** bacteriophage (phage) therapy, phage BUCT541, MDR-KP K1-ST23, *Galleria mellonella*, BALB/c mice

## Abstract

Over the past decades, the spread of multi-drug-resistant *Klebsiella pneumoniae* (MDR-KP) is becoming a new threat and new effective therapies against this pathogen are needed. Bacteriophage (phage) therapy is considered to be a promising alternative treatment for MDR-KP infections compared with antibacterial drug usage. Here, we reported a new phage BUCT541 which can lyse MDR-KP ST23. The genome of BUCT541 is a double-stranded linear 46,100-bp long DNA molecule with 48% GC content through the Next generation sequencing (NGS) data. A total of 81 open reading frames and no virulence or antimicrobial resistance genes are annotated in the BUCT541 genome. BUCT541 was able to lyse 7 of the 30 tested MDR-KP according to the host range analysis. And the seven sensitive strains belonged to the *K. pneumoniae* K1-ST23. BUCT541 exhibited high thermal stability (4–70°C) and broad pH tolerance (pH 3-11) in the stability test. The *in vivo* results showed that BUCT541 (4 × 10^5^ plaque-forming units (PFU)/each) significantly increased the survival rate of *K. pneumoniae* infected *Galleria mellonella* from 5.3% to 83.3% within 48 h. Moreover, in the mouse lung infection model, high doses of BUCT541 (2 × 10^7^ PFU/each) cured 100% of BALB/c mice that were infected with *K. pneumoniae*. After 30 h of treatment with phage BUCT541 of the multiplicity of infection (MOI) = 10, the *K. pneumoniae* in the lungs of mice was lower than 10^4^ CFU/mL, compared to the control group 10^9^ CFU/mL. Together, these findings indicate that phage BUCT541 holds great promise as an alternative therapy with excellent stability and a wide lysis range for the treatment of MDR-KP ST23 infection.

## Introduction

*Klebsiella pneumoniae* is an opportunistic hospital-acquired pathogen that causes serious hospital infections, especially in immunocompromised patients (Calfee, 2010; Wang et al., 2020). As the second-ranked hospital-acquired pathogen, *K. pneumoniae* can parasitize the intestines, lungs, and skin (Rees et al., 2016; Marques et al., 2019; Zaki, 2019) to cause serious infections such as urinary tract infections, lung infections, bloodstream infections, and sepsis (Mazzariol et al., 2017; Ishiguro et al., 2020). The β-lactam antibiotics are used for *K. pneumoniae* infections, but with the prevalence of extended-spectrum-lactamases (ESBLs)-producing *K. pneumoniae*, carbapenems have become first-line antibiotics to treat *K. pneumoniae* infection (Grillon et al., 2016; Mikhail et al., 2019). In 1999, the carbapenem-resistant *K. pneumoniae* (CR-kpn) strain was first observed (Koh et al., 1999). From then on, CR-kpn with New Delhi metallo-β-lactamase (NDM) and *Klebsiella pneumoniae* carbapenemase (KPC) disseminated worldwide, which makes the treatment options limited to a few antibiotics such as tigecycline and polymyxins (Motsch et al., 2020). However, in a recent study, Van Duin et al. demonstrated that 46% of CR-KPN are also resistant to tigecycline to some extent, and as the last resort drug, polymyxins had a big safety concern for its nephrotoxicity and neurotoxicity (van Duin et al., 2015). Moreover, the emergence of carbapenemase-resistant hypervirulent *K. pneumoniae* (CR-hvKP) in recent years, which caused a higher mortality rate, has further exacerbated the dilemma of antibiotic therapy, triggering the need for alternative therapies (Lan et al., 2021).

Bacteriophages (phages) are bacterial viruses that specifically recognize, infect, and replicate within host bacteria. Phages have been considered therapeutic agents since the early 1920s as a result of their unique antibacterial ability (Wang et al., 2021). In addition, phages have the advantages of strong antibacterial ability and high quantity, as well as low toxic side effects to humans, and are considered to be the most promising drugs to replace traditional antibiotics. Some studies using mice as an animal model have shown that phages have promising therapeutic effects on pneumonia, liver abscesses, and burn infections caused by *K. pneumoniae* (Lin et al., 2014; Chadha et al., 2017; Anand et al., 2020). In addition, phage therapy has also been used in clinical practice to cure serious infections caused by MDR-KP. For example, in 2019, Wu Nannan et al. have reported the combination of phage and antibiotics against *K. pneumoniae* to cure a recurrent urinary tract infection caused by MDR-KP (Bao et al., 2020). In February 2019, a 62-year-old patient, with a prosthetic joint infection caused by *K. pneumoniae* KpJH46, was successfully treated with phage KpJH46Φ2 combined with antibiotics at the Mayo Clinic Infectious Disease Unit in Rochester, MN, USA (Cano et al., 2021). Although phage therapy has great potential for future applications, the specificity of phages to bacteria and the clearance of phages by the immune system are challenges for phage therapy. The discovery of new phages and evaluation of their antimicrobial capacity are one of the effective ways to overcome these challenges.

Here, we reported a lytic phage BUCT541 against *K. pneumoniae*. Not only the physiological characteristics of phage BUCT541 were determined but also the genetic background of BUT541 was revealed using bioinformatics tools. In addition, the ability of phage BUCT541 to treat *K. pneumoniae* infection was further evaluated in the *Galleria mellonella* larvae infection model and the mice infection model.

## Materials and methods

### Isolation and purification of BUCT541

Bacteriophage BUCT541 was isolated by using *K. pneumoniae* S-2007 as the host strain from sewage samples, which were collected from the Aviation General Hospital sewer system. The isolation method of BUCT541 was similar to the one described by Li et al. with slight variations (Li et al., 2021). In brief, untreated sewage samples were centrifuged to remove large impurities. Then, the samples were concentrated using a permeable membrane and PEG8000 for 3 h, and the concentrated solution was filtered through a 0.22-μm filter. Concentrated sewage samples (2 μL) were spotted on the lawn with *K. pneumoniae* S-2007 and cultured overnight at 37°C to obtain phage plaques of BUCT541. Plaques were picked up and co-cultured with *K. pneumoniae* S-2007 in LB medium for 8 h. The supernatant of the culture after centrifuging was filtered through a 0.22-μm filter and purified the phage BUCT541 by the double-layer plate method until a single morphology phage plaque was formed on the lawn with *K. pneumoniae* S-2007. And then, the phage BUCT541 suspension was further purified by discontinuous cesium chloride (CsCl) density gradient (ρ = 1.3, 1.5, and 1.7) and centrifuged at 30,000 ×*g* for 2 h at 4°C. Finally, the banded phage particle was collected and dialyzed with PBS buffer (0.1 M, pH 7.4) (Uchiyama et al., 2011).

### Electron micrograph of phage BUCT541

To visualize phages, the 30 μL of purified phages BUCT541 lysate was incubated with the carbon-coated copper grid for 10 min and stained with 2% uranyl acetate for 90 s and subsequently air dried. The morphology of the phages was examined with a transmission electron microscope (JEM-1200EX, Japan) at 80 kV.

### Multilocus sequence typing (MLST) and capsule type of bacteria

Thirty strains of MDR-KP were collected from the Aviation General Hospital. The lytic range of phage BUCT541 was determined by the double-layer plate method and spotting method. Some phages have been reported to have the ability to lyse different subtypes of the same bacteria (Zhang et al., 2021). To investigate whether phage BUCT541 can lyse different subtypes of *K. pneumoniae*, the MLST and capsule type of 30 strains of MDR-KP were identified. In brief, seven housekeeping genes (*rpoB, gapA, mdh, pgi, phoE, infB*, and *tonB*) and *wzi* gene of the 30 bacteria were subjected to PCR amplification. The amplified products were sent to the Beijing Ruibo Xingke Biotechnology Co., Ltd. for bidirectional sequencing. The sequencing results were analyzed by the MLST database (https://bigsdb.pasteur.fr/cgi-bin/bigsdb/bigsdb.pl?db=pubmlst_klebsiella_seqdef&l=1) for analysis. Primer sequences were shown in Supplementary Table S1.

### Optimal multiplicity of infection of BUCT541

S-2007 was cultured to exponential phase and the number was counted. Around 100 μL of the mixture including BUCT541 and S-2007 with different MOIs (0.001, 0.01, 0.1, 1, 10, 100) were added to the 10 mL LB medium for overnight culture at 37°C with shaking (220 rpm). Then, the phage titers were determined after being cultured. The proportion with the highest phage titer was the optimal multiplicity of infection.

### One-step growth curve assay of phage BUCT541

The mixture of BUCT541 and host S-2007 was incubated for 10 min at room temperature with optimal MOI. The supernatant was discarded after centrifugation at 4°C for 3 min at 12,000 ×*g*. The precipitate was resuspended with LB and centrifuged for 3 min at 4°C and 12,000 ×*g*, and then the above steps are repeated. The mixture after resuspension was added to 25 mL of LB liquid medium and cultured at 37°C for 150 min with shaking at 200 rpm. The titer of phage BUCT541 was detected at different time points. The above experiments were performed on ice. The titer of BUCT541 at different time points was detected by the double-layer plate method.

### The stability determination of phage BUCT541

The method of phage BUCT541 stability determination is similar to that described by Ahmed R. Sofy et al. but with slight changes (Sofy et al., 2021). About 500 μL (3 × 10^8^ PFU/mL) of phage BUCT541 was incubated at 4, 37, 50, 60, and 70°C for 0.5, 1, 1.5, and 2 h, respectively. The titer of phage BUCT541 was detected by the double-layer plate method with different incubation times. Similarly, 500 μL (5 × 10^7^ PFU/mL) of phage BUCT541 was incubated for 2, 4, and 6 h at pH = 3, 5, 7, 9, and 11, respectively. The titer of phage BUCT541 was detected with different incubation times.

### Genome sequence and bioinformatics analysis of phage BUCT541

Extract the phage genome using the classical K/SDS method and the phage DNA samples were sent to Annoroad company for next-generation sequencing (NGS). The raw sequenced reads were assessed for quality using FastQC v.0.11.5 and filtered for low-quality reads and adapter regions using Trimmomatic v.0.36 (Madaha et al., 2020). The high-quality reads were spliced using SPAdes v3.13.0 (Zoledowska et al., 2018). Spliced data were removed from head and tail duplicate regions by SnapGene. Genome sequence similarity was aligned using BLASTn (https://blast.ncbi.nlm.nih.gov/Blast.cgi?PROGRAM=blastn&PAGE_TYPE=BlastSearch&LINK_LOC=blasthome). Use the online website to predict RAST open reading frames (ORFs) (https://rast.nmpdr.org/rast.cgi) and rectify the predictions through the NCBI database. The molecular weight of ORFs encoded proteins was determined using the ExPASY ProtParam online website (https://web.expasy.org/protparam/) (Karunarathna et al., 2020). Gene function maps were created using a lab-built program and retouched with the software Inkscape 0.92.3.0. The tRNAscan-SE v.2.0 was used to predict tRNA (http://lowelab.ucsc.edu/cgi-bin/tRNAscan-SE2.cgi) (Alam and Rao, 2008). ResFinder (https://cge.cbs.dtu.dk/services/ResFinder/) and VirulenceFinder (https://cge.cbs.dtu.dk/services/VirulenceFinder/) were used to detect drug resistance genes and virulence genes, respectively (Joensen et al., 2014; Bortolaia et al., 2020). Construction of a complete genome phylogenetic tree based on the whole genome sequence of BUCT541 using the genome-BLAST distance phylogenetic approach in virus classification and VICTOR (https://ggdc.dsmz.de/submit_victor_job.php). Phylogenetic tree of large terminase and minor-capsid proteins were constructed by Neighbor joining method (NJ method). And Bootstrap method was used to check the phylogenetic tree. In addition, the complete genome sequence of phage BUCT541 was aligned with other phages using the BLASTn tool in the NCBI database. Complete genome sequence similarity between BUCT541 and other phages was visualized by the Circoletto program (Darzentas, 2010) (http://tools.bat.infspire.org/Circoletto/).

### Assessment of the efficacy of BUCT541 against *K. pneumoniae* S-2007 *in vitro*

The *K. pneumoniae* S-2007 was cultured to OD_600_ of about 0.25 at 37°C and 120 rpm. The 5 mL of phage BUCT541 with different titers (10^5^ PFU/mL, 10^6^ PFU/mL, 10^7^ PFU/mL, and 10^8^ PFU/mL) were mixed with 25 mL S-2007, respectively. The mixture of different MOIs (0.01, 0.1, 1, 10) was continued to cultivate at 37°C, 120 rpm for 8 h. For the control group, only 5 mL LB liquid medium and 25 mL S-2007 were mixed and cultured for 8 h at 37°C, 120 rpm. And the concentration of *K. pneumoniae* S-2007 during the cultivation was responded by OD_600_. OD_600_ was measured by nanodrop (Thermo scientific).

### Therapeutic effect of phage BUCT541 in the *G. mellonella* larvae

The *G. mellonella* larvae (Huiyude Biotech Company, Tianjin, China) were selected with a length of 25 ± 5 mm, weight of 300 ± 50 mg, strong activity, and no black patches on the body surface. Thirty larvae were used as a sample population for every group. *K. pneumoniae* S-2007 were grown in LB and harvested in exponential phase. After being washed with PBS, 4 μL 1 × 10^8^ CFU/mL S-2007 (4 × 10^5^ CFU/each) was injected into the last right proleg of larvae by a micro-sample syringe. And half an hour after infection, phage BUCT541 with different titers (1 × 10^9^ PFU/mL, MOI = 10; 1 × 10^8^ PFU/mL, MOI = 1; 1 × 10^7^ PFU/mL, MOI = 0.1) was injected from the last left proleg of larvae for treatment. The number of surviving *G. mellonella* larvae at different MOI groups was observed and recorded every 2 h for 48 h. The positive control group was treated with PBS (4 μL/each), and only PBS or phage BUCT541 (1 × 10^9^ PFU/mL, 4 μL/each) was injected as a negative group. Results were considered valid when all the PBS-injected larvae survived during the experiment.

### Therapeutic effect of phage BUCT541 in the mouse model

Female BALB/c mice were purchased from the SPF (Beijing, China) Biotechnology Co., Ltd., aged 3–5 weeks, and weighed 18–20 g. Mice were fed with sterile water and chow for 4 days and their health was observed daily. Thirteen BALB/c mice were used as a sample population in every group, which were infected with 20 μL 1 × 10^8^ CFU/mL S-2007 (2 × 10^6^ CFU/each) by nasal drip. And 6 h after infection, 20 μL phage BUCT541 with different titers (1 × 10^9^ PFU/mL, MOI = 10; 1 × 10^8^ PFU/mL, MOI = 1; 1 × 10^7^ PFU/mL, MOI = 0.1; 1 × 10^6^ PFU/mL, MOI = 0.01) was used to treat BALB/c mice by nasal drip. The number of surviving mice in different MOI groups was observed and recorded every 24 h for 7 days. The positive group was treated with 20 μL PBS. The negative control group was treated with 20 μL phage BUCT541 (1 × 10^9^ PFU/mL) and PBS.

To further investigate the effect of phage BUCT541 against *K. pneumoniae* S-2007 in the lungs of BALB/c mice, the number of *K. pneumoniae* S-2007 and the pathological changes in the lungs of mice were examined after 30 h of phage treatment. In brief, after 30 h of phage treatment, three mice were randomly selected from 13 mice for dissection. A portion of the lung from each mouse was made into a specimen for pathological section analysis. The other part of the lung was ground into homogenate and the number of *K. pneumoniae* S-2007 was determined by the plating method.

### Data availability

All data were analyzed using the GraphPad Prism 8.0.1 and expressed as means and standard deviation values. Student's test (*t* test) analysis was used in **Figures 2A**, **7C**. The complete genome sequence of bacteriophage BUCT541 has been deposited in GenBank under the accession number MZ836210.1

## Results

### Morphology and host range of phage BUCT541

**Figure 1:**
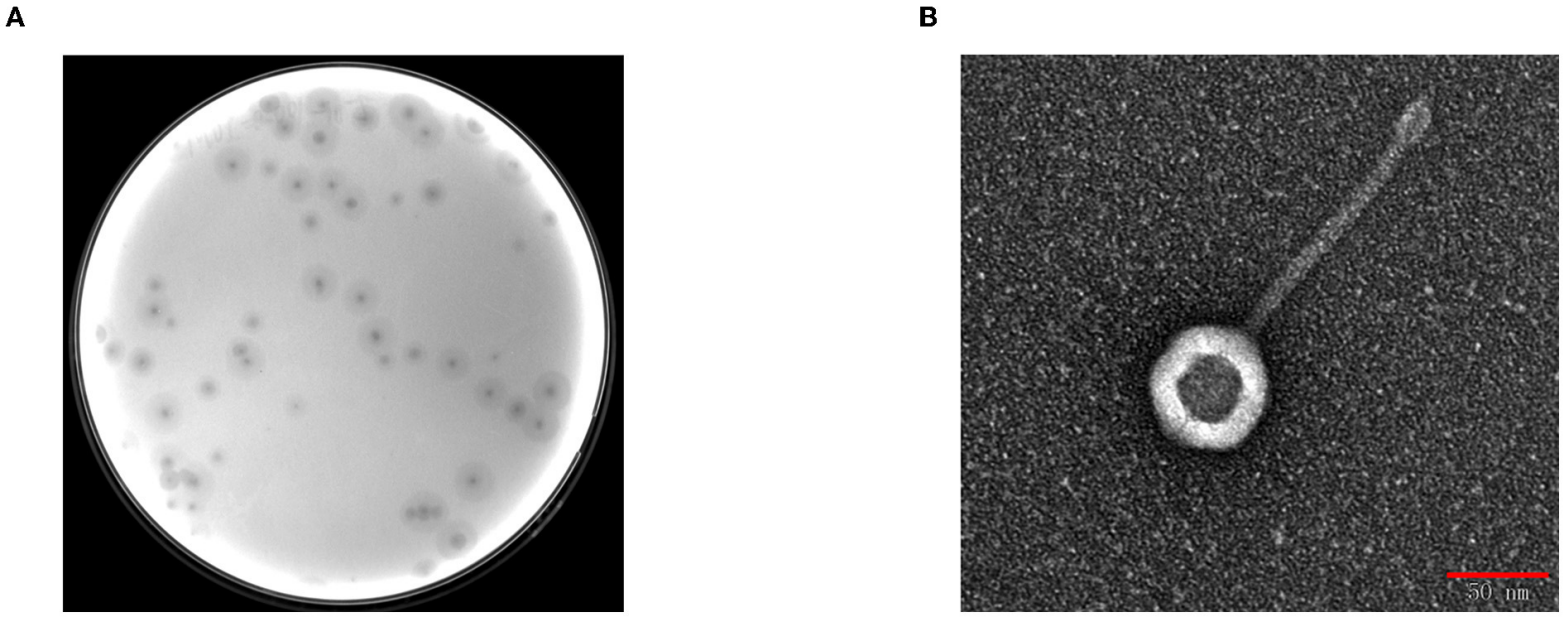
Plaques morphology of bacteriophage BUCT541. **(A)** Phage plaques formed of BUCT541 on the lawn with *K. pneumoniae* S-2007; **(B)** Transmission electron micrograph image of BUCT541.

BUCT541 forms clear, translucent, and regular plaques on a lawn of *K. pneumoniae* S-2007 with clear haloes distributed around the plaque center (Figure 1A). The electron micrographs show that the head diameter of BUCT541 was 57.013 ± 1.302 nm and the tail length was 147.263 ± 2.349 nm (Figure 1B). Based on the morphological classification, BUCT541 was considered to belong to the *Siphoviridae* family. Thirty strains of *K. pneumoniae* were collected from the Aviation General Hospital, and seven of them could be lysed by BUCT541.

### Multilocus sequence typing and capsular type identification of bacteria

Seven alleles of *gapA, infB, mdh, pgi, phoE, rpoB* and *tonB* were arranged in that order and analyzed using the multilocus sequence typing scheme that was developed for *K. pneumoniae*. According to the analysis of the MLST Database, the capsular type and multi-locus sequence type of host bacteria of phage BUCT541 belong to K1 and sequence type 23 (ST23), respectively. K1-ST23 *K. pneumoniae* is one of the hypervirulent *K. pneumoniae* (hv-KP) in clinical practice (Nakamura et al., 2021). The capsular type and multi-locus sequence type of other *K. pneumoniae* were shown in Table 1.

**Table 1:** Detail information of bacteria of host range tested.

**Species**	**Strain**	**ST type**	**Capsular type**	**Origin**	**Sensitivity**
*K. pneumoniae*	S-2007	ST23	K1	Aviation General Hospital	+
*K. pneumoniae*	081	ST23	K1	Aviation General Hospital	+
*K. pneumoniae*	064	ST23	K1	Aviation General Hospital	+
*K. pneumoniae*	K7	ST23	K1	Aviation General Hospital	+
*K. pneumoniae*	2,752	ST23	K1	Aviation General Hospital	+
*K. pneumoniae*	2,755	ST23	K1	Aviation General Hospital	+
*K. pneumoniae*	2,024	ST23	K1	Aviation General Hospital	+
*K. pneumoniae*	2,011	N/A	K25	Aviation General Hospital	–
*K. pneumoniae*	2,012	ST11	K25	Aviation General Hospital	–
*K. pneumoniae*	2,013	ST15	K19	Aviation General Hospital	–
*K. pneumoniae*	2,014	N/A	K47	Aviation General Hospital	–
*K. pneumoniae*	2,015	ST11	K47	Aviation General Hospital	–
*K. pneumoniae*	2,086	ST11	K64	Aviation General Hospital	–
*K. pneumoniae*	2,017	ST15	K19	Aviation General Hospital	–
*K. pneumoniae*	2,018	ST11	N/A	Aviation General Hospital	–
*K. pneumoniae*	2,019	N/A	K47	Aviation General Hospital	–
*K. pneumoniae*	2,020	N/A	K47	Aviation General Hospital	–
*K. pneumoniae*	2,068	ST15	K19	Aviation General Hospital	–
*K. pneumoniae*	2,022	ST11	K47	Aviation General Hospital	–
*K. pneumoniae*	2,002	ST15	K19	Aviation General Hospital	–
*K. pneumoniae*	2,003	ST15	K19	Aviation General Hospital	–
*K. pneumoniae*	2,004	ST11	K47	Aviation General Hospital	–
*K. pneumoniae*	2,005	ST11	K25	Aviation General Hospital	–
*K. pneumoniae*	2,006	ST11	K47	Aviation General Hospital	–
*K. pneumoniae*	2,008	ST11	K64	Aviation General Hospital	–
*K. pneumoniae*	2,009	ST11	K64	Aviation General Hospital	–
*K. pneumoniae*	2,021	N/A	K64	Aviation General Hospital	–
*K. pneumoniae*	2,022	ST11	K47	Aviation General Hospital	–
*K. pneumoniae*	2,026	ST11	K64	Aviation General Hospital	–
*K. pneumoniae*	2,027	ST15	K19	Aviation General Hospital	–

“+”* susceptible; “–” resistance; “N/A” mutation*.

### Physiological characterization of phage BUCT541

**Figure 2:**
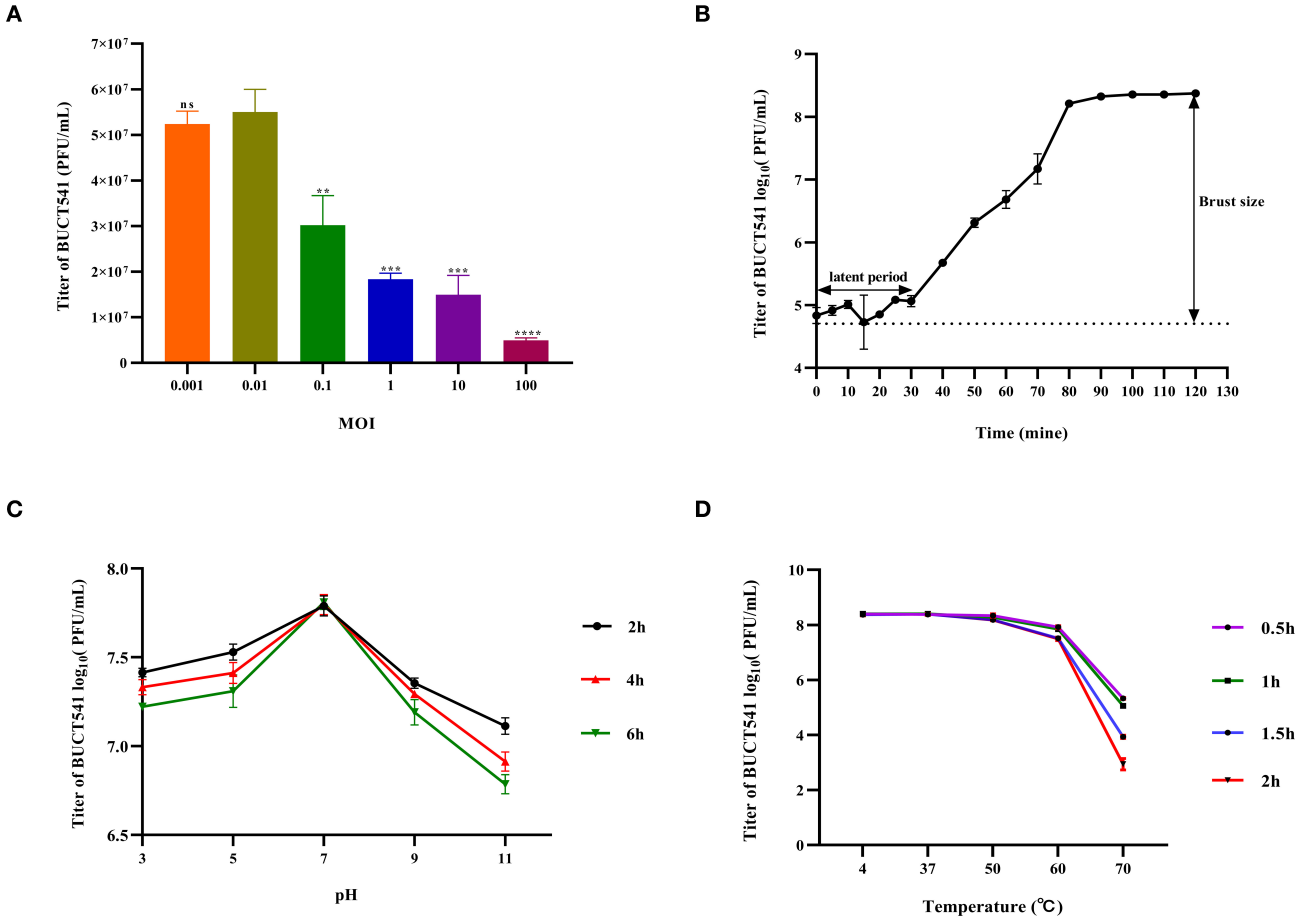
Physiological characterization of phage BUCT541. **(A)** Optimal MOI assays of bacteriophage BUCT541 (****P < 0.0001, ***P < 0.001, and **P < 0.01 indicate a significant difference compared to the MOI 0.01); **(B)** One-step growth curve of bacteriophage BUCT541; **(C)** pH stability of bacteriophage BUCT541; **(D)** Thermal stability of BUCT541.

When the MOI was 0.01, the titer of phage BUCT541 in the culture was significantly higher than other MOIs, indicating that the MOI of 0.01 was most suitable for the growth of phage BUCT541 (Figure 2A). The one-step growth curve showed that the latent and lytic periods of BUCT541 were about 30 min and 50 min, respectively (Figure 2B). The titer of BUCT541 was highest at pH = 7 and decreased significantly at pH = 9 and pH = 11 with the same incubation time. In the same buffer, the titer of BUCT541 decreased at the longer incubation times, except at pH = 7 where the titer was essentially constant at all incubation times tested (Figure 2C). We also found that the titer of BUCT541 was significantly less in the acidic buffer than that in the alkaline buffer, indicating that BUCT541 was more stable in the acidic environments. The titer of BUCT541 was relatively stable at 4 to 60°C but rapidly decreased at 70°C (Figure 2D), indicating BUCT541 had good thermal stability.

### Characteristics and annotations of the complete genome of phage BUCT541

**Figure 3:**
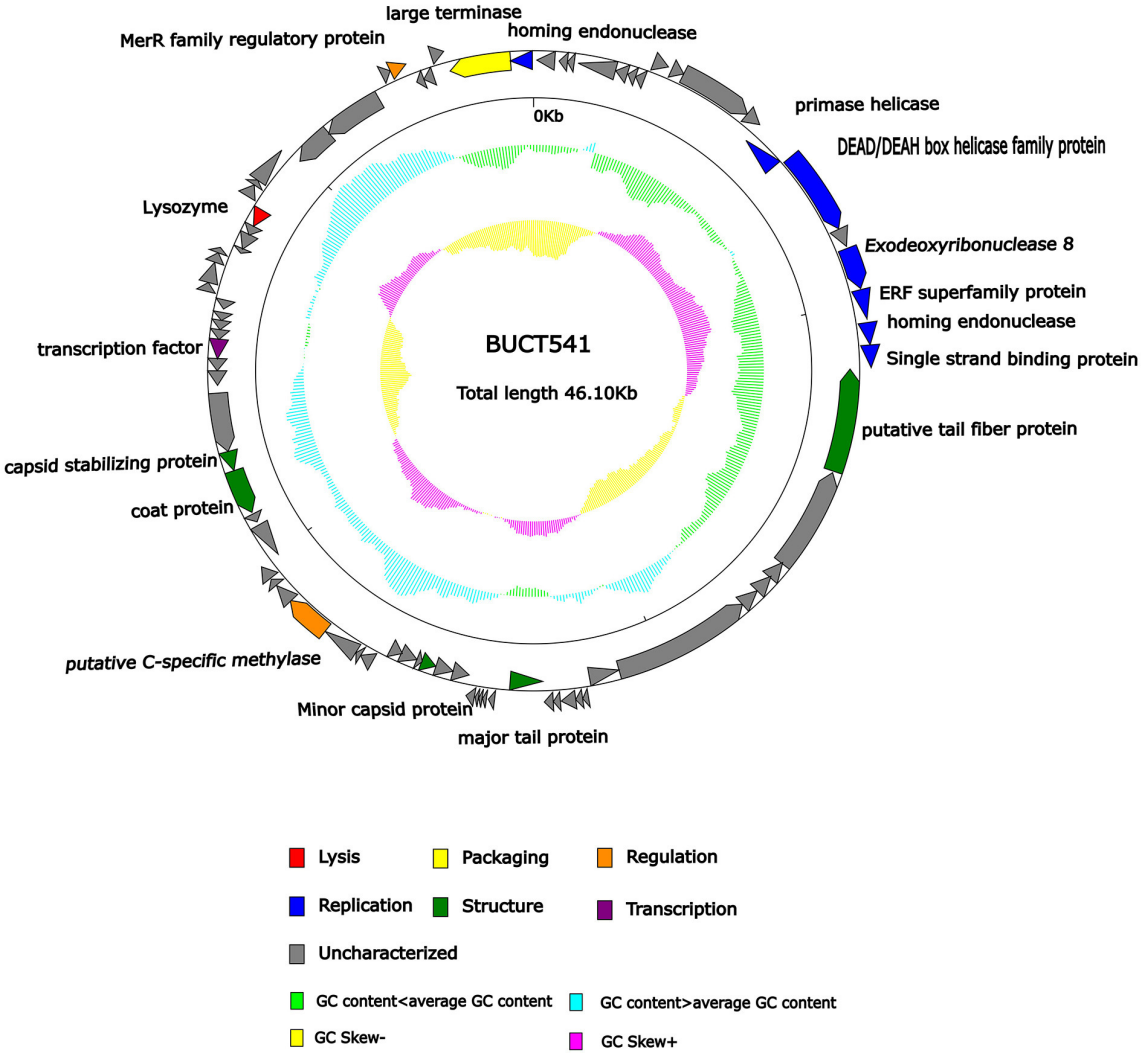
Genomic map of bacteriophage BUCT541 and its genetic characteristics. Open reading frames (ORFs) are represented in different colors according to their functional categories.

**Figure 4:**
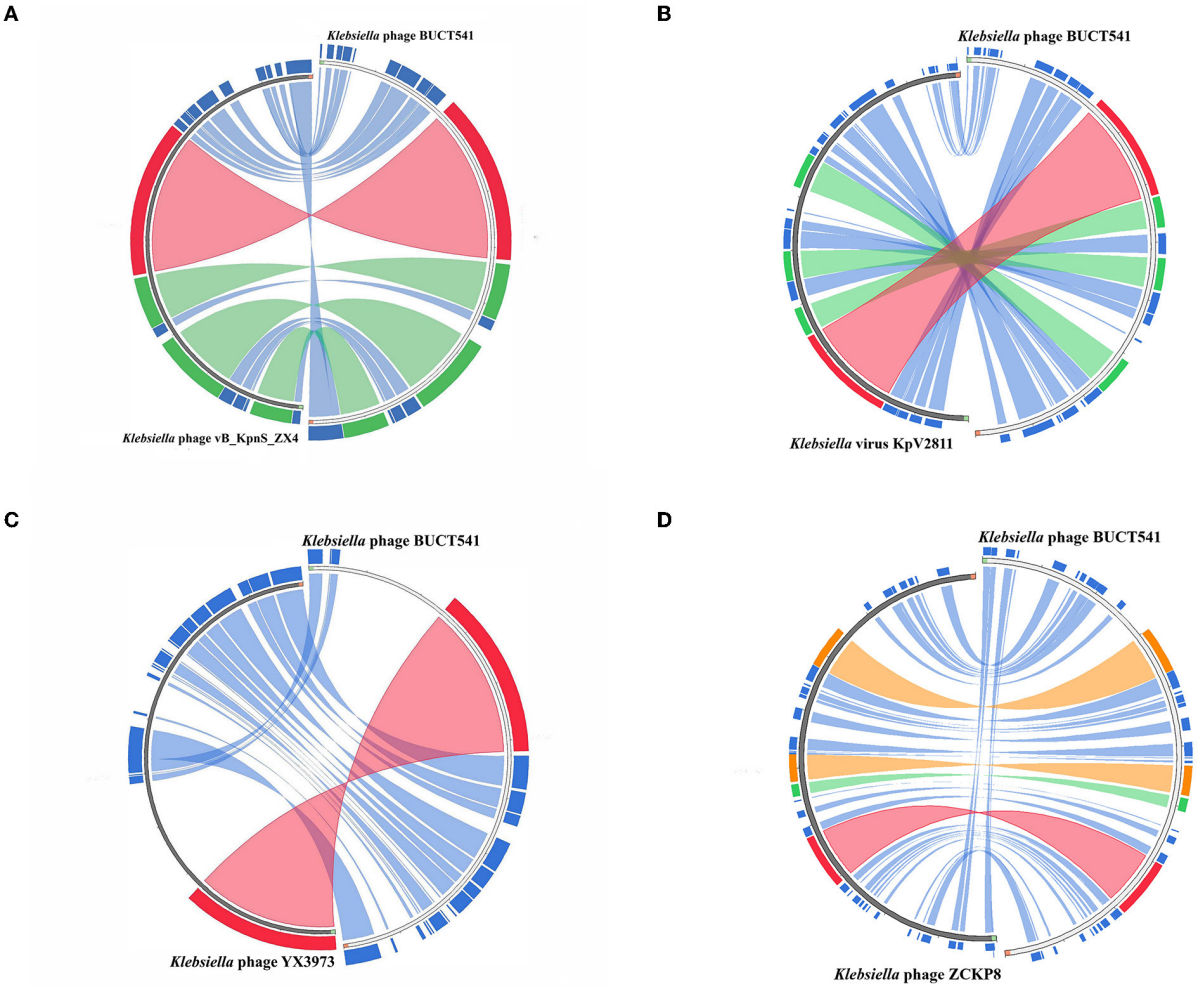
Circos plot depicting sequence similarities of *Klebsiella* phage BUCT541 with **(A)** *Klebsiella phage* vB_KpnS ZX4, **(B)** *Klebsiella* virus KpV2811, **(C)** *Klebsiella* phage YX3973, and **(D)** *Klebsiella* phage ZCKP8. The red color signifies a high sequence similarity followed by orange, green, and blue. Ratio coloring with blue ≤ 0.25, green ≤ 0.50, orange ≤ 0.75, and red > 0.75.

**Figure 5:**
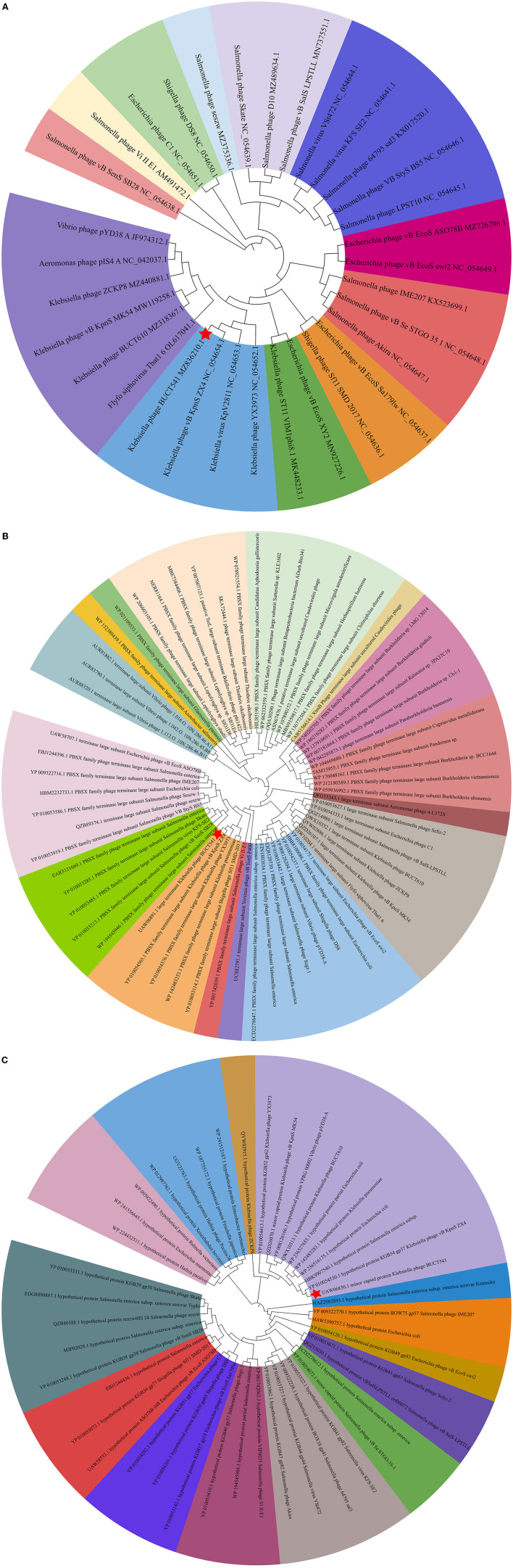
**(A)** Phylogenetic relations of *Klebsiella* phage BUCT541 based on the whole genome sequence generated by VICTOR; **(B)** Neighbor-joining tree of *Klebsiella* phage BUCT541 compared to other phages available in GenBank based on the large terminase alignment of amino acid sequences; **(C)** Neighbor-joining tree of *Klebsiella* phage BUCT541 compared to other phages available in the GenBank based on the alignment of minor-capsid amino acid sequences.

The complete genome sequence of phage BUCT541 has been submitted to NCBI (GenBank: MZ836210.1). The BUCT541 genome is a double-stranded linear 46,100 bp long DNA molecule with 48% GC content. A total of 81 open reading frames (ORFs) are annotated (Table 2) and 21 of them encode proteins with functions associated with phage lysis, regulation, packaging, structure, and replication (Figure 3). The other ORFs are annotated as encoding hypothetical proteins. The genome sequence alignment of phage BUCT541 with the genome sequences of other *Klebsiella* phages showed that BUCT541 shared the highest cover (84%) and identity (97.9%) with *Klebsiella* phage vB_KpnS_ZX4 (GenBank: NC_054654.1) (Figure 4A), followed by *Klebsiella* virus KpV2811 (GenBank: NC_054653.1) (Figure 4B), *Klebsiella* phage YX3973 (GenBank: NC_054652.1) (Figure 4C), and *Klebsiella* phage ZCKP8 (GenBank: MZ440881.1) (Figure 4D), which shared 71%, 64%, and 60% identity, respectively. In addition, the complete genome phylogenetic tree of BUCT541 and other phages showed that BUCT541 also shared a close evolutionary relationship with *Vibrio* phage pYD38-A (GenBank: JF974312.1) and *Aeromonas* phage pIS4-A (GenBank: NC_042037.1) (Figure 5A).

**Table 2:** Annotated ORFs in the genome of phage BUCT541.

**ORF**	**Strand**	**Start**	**Stop**	**Length (AA)**	**Putative function**	**Best-match BLASTp result**	**Query cover (%)**	**E-values**	**Identity (%)**	**Accession**	**MW (kDa)**
ORF1	–	489	73	138	Hypothetical protein	*Salmonella enterica*	100	2e-91	99.28	WP_194545944.1	15.5
ORF2	–	785	591	64	Hypothetical protein	Klebsiella phage YX3973	100	2e-37	95.31	YP_010054373.1	7.1
ORF3	–	943	785	52	Hypothetical protein	*Siphoviridae* sp.	100	7e-30	98.08	DAE56162.1	6.1
ORF4	–	1,919	1,035	294	Hypothetical protein	Klebsiella phage vB_Kpns_ZX4	70	8e-110	77.78	YP_010054560.1	32.1
ORF5	–	2,204	1,926	92	Hypothetical protein	Klebsiella virus KpV2811	100	1e-58	100	YP_010054492.1	10.5
ORF6	–	2,405	2,220	61	Hypothetical protein	*Siphoviridae* sp.	100	5e-37	100	DAF31840.1	6.7
ORF7	–	2,617	2,408	69	Hypothetical protein	Not hit	–	–	–	–	7.9
ORF8	+	2,679	3,014	111	Hypothetical protein	*Siphoviridae* sp.	100	1e-49	76.58	DAE56151.1	12.5
ORF9	+	3,103	3,381	92	Hypothetical protein	*Siphoviridae* sp.	100	5e-60	95.65	DAE56150.1	10.5
ORF10	+	3,381	5,030	549	Hypothetical protein	*Siphoviridae* sp.	100	0.0	97.81	DAE56149.1	61.2
ORF11	+	5,027	5,383	118	Hypothetical protein	Not hit	–	–	–	–	13.4
ORF12	–	6,290	5,397	297	TPA: MAG TPA: primase helicase	*Siphoviridae* sp.	100	0.0	95.62	DAF74631.1	33.5
ORF13	+	6,365	8,275	636	DEAD/DEAH box helicase family protein	*Klebsiella pneumoniae*	100	0.0	98.27	WP_142483293.1	72.3
ORF14	+	8,286	8,702	138	Hypothetical protein	Klebsiella phage BUCT610	100	2e-96	96.38	QWX10336.1	15.7
ORF15	+	8,747	9,685	312	TPA: MAG TPA: Exodeoxyribonuclease 8	*Siphoviridae* sp.	100	0.0	96.79	DAN88330.1	35.6
ORF16	+	9,709	10,371	220	TPA: MAG TPA: ERF superfamily protein	*Siphoviridae* sp.	100	4e-160	100	DAE85886.1	24.0
ORF17	+	10,452	10,952	166	TPA: MAG TPA: homing endonuclease	*Siphoviridae* sp.	100	5e-118	100	DAE85905.1	18.7
ORF18	+	10,952	11,455	167	TPA: MAG TPA: Single strand binding protein	*Siphoviridae* sp.	100	4e-111	95.21	DAE85903.1	18.5
ORF19	–	13,937	11,484	817	Hypothetical protein	Klebsiella virus KpV2811	100	0.0	98.04	YP_010054425.1	86.3
ORF20	–	16,432	13,976	818	Hypothetical protein	Klebsiella phage vB_Kpns_ZX4	100	0.0	99.76	YP_010054545.1	89.6
ORF21	–	16,862	16,404	152	Hypothetical protein	Klebsiella phage vB_Kpns_ZX4	100	1e-109	99.34	YP_010054544.1	17.5
ORF22	–	17,295	16,825	156	Hypothetical protein	Klebsiella phage YX3973	100	4e-110	99.36	YP_010054357.1	17.9
ORF23	–	17,765	17,295	156	Hypothetical protein	Klebsiella phage YX3973	100	1e-111	100	YP_010054356.1	17.8
ORF24	–	21,046	17,765	1,093	Tail length tape-measure protein 1	Klebsiella phage vB_Kpns_ZX4	100	0.0	99.82	YP_010054541.1	114.3
ORF25	–	21,753	21,046	235	Hypothetical protein	*Siphoviridae* sp.	100	1e-172	99.15	DAF78471.1	26.4
ORF26	+	21,850	21,978	42	Hypothetical protein	Klebsiella phage vB_Kpns_ZX4	100	3e-20	100	YP_010054539.1	4.7
ORF27	+	21,981	22,133	50	Hypothetical protein	Klebsiella phage vB_KpnS_ZX4	100	2e-27	100	YP_010054538.1	5.6
ORF28	+	22,133	22,450	105	Hypothetical protein	Klebsiella phage YX3973	100	9e-73	100	YP_010054353.1	12.1
ORF29	+	22,469	22,621	50	Hypothetical protein	*Siphoviridae* sp.	100	3e-26	98.00	DAF31827.1	5.6
ORF30	+	22,624	22,818	65	Hypothetical protein	*Siphoviridae* sp.	100	1e-38	100	DAF31826.1	7.9
ORF31	-	23,594	22,839	251	TPA: MAG TPA: major tail protein	*Siphoviridae* sp.	100	3e-172	95.22	DAE85900.1	26.8
ORF32	+	23,904	24,038	44	Hypothetical protein	Enterobacter phage Tyrion	90	4e-14	87.50	YP_009447826.1	4.8
ORF33	+	24,081	24,165	–	tRNA-Ser-GCT	–	–	–	–	–	–
ORF34	+	24,175	24,248	–	tRNA-Arg-TCT	–	–	–	–	–	–
ORF35	+	24,255	24,328	–	tRNA-Met-CAT	–	–	–	–	–	–
ORF36	+	24,347	245,32	61	Hypothetical protein	Klebsiella virus KpV2811	100	9e-36	96.72	YP_010054438.1	7.0
ORF37	–	24,940	24,557	127	Hypothetical protein	Vibrio phage pYD38-A	100	6e-89	100	YP_008126240.1	14.4
ORF38	–	25,353	24,937	138	Hypothetical protein	Klebsiella virus KpV2811	100	1e-94	99.28	YP_010054440.1	15.0
ORF39	–	25,701	25,357	114	TPA: MAG TPA: Minor capsid protein	*Siphoviridae* sp.	100	4e-79	100	DAF31775.1	12.4
ORF40	–	25,823	25,689	44	Hypothetical protein	Klebsiella virus KpV2811	100	1e-21	100	YP_010054442.1	5.1
ORF41	–	26,212	25,820	130	Hypothetical protein	Klebsiella phage YX3973	100	2e-89	99.23	YP_010054412.1	13.7
ORF42	–	26,477	26,190	95	Hypothetical protein	Klebsiella phage YX3973	100	5e-62	100	YP_010054411.1	10.1
ORF43	+	26,708	27,001	97	Hypothetical protein	*Siphoviridae* sp.	100	2e-64	100	DAF31817.1	10.5
ORF44	+	26,998	27,114	38	Hypothetical protein	Klebsiella phage vB_KpnS_ZX4	100	8e-17	100	YP_010054525.1	4.3
ORF45	+	27,122	27,937	271	Hypothetical protein	Klebsiella phage YX3973	100	0.0	100	YP_010054409.1	29.8
ORF46	+	27,937	28,959	340	Putative C-specific methylase	Klebsiella phage vB_KpnS_ZX4	100	0.0	88.82	YP_010054523.1	37.3
ORF47	+	28„943	29,401	152	Hypothetical protein	Klebsiella phage YX3973	100	2e-107	99.34	YP_010054407.1	17.9
ORF48	+	29,412	29,585	57	Hypothetical protein	Klebsiella phage BUCT610	100	4e-34	98.25	QWX10306.1	6.7
ORF49	+	29,611	29,931	106	Hypothetical protein	*Siphoviridae* sp.	100	5e-66	91.51	DAN88390.1	12.7
ORF50	–	30,767	29,946	273	Hypothetical protein	*Siphoviridae* sp. cthqG28	94	1e-180	98.44	DAD75110.1	29.0
ORF51	–	31,060	30,851	69	Hypothetical protein	Klebsiella phage vB_KpnS_ZX4	100	5e-40	100	YP_010054519.1	8.0
ORF52	–	32,175	31,102	357	Coat protein	Klebsiella phage vB_KpnS_ZX4	100	0.0	98.88	YP_010054518.1	39.8
ORF53	–	32,648	32,175	157	TPA: MAG TPA: capsid stabilizing protein	*Siphoviridae* sp.	100	1e-109	100	DAE85894.1	16.2
ORF54	–	34,048	32,648	466	TPA: MAG TPA: protein of unknown function	*Siphoviridae* sp.	100	0.0	99.14	DAF31811.1	51.4
ORF55	–	34,575	34,231	114	Hypothetical protein	Klebsiella phage BUCT610	100	1e-75	94.74	QWX10298.1	13.0
ORF56	–	34866	34576	96	Hypothetical protein	*Klebsiella* phage vB_KpnS_ZX4	100	1e-64	100	YP_010054514.1	11.0
ORF57	–	35,324	34,854	156	TPA: MAG TPA: transcription factor	*Siphoviridae* sp.	100	2e-111	100	DAF31807.1	17.7
ORF58	–	35,550	35,314	78	Hypothetical protein	Klebsiella phage vB_KpnS_ZX4	100	1e-49	98.72	YP_010054512.1	8.7
ORF59	–	35,771	35,547	74	Hypothetical protein	Klebsiella phage vB_KpnS_ZX4	100	1e-46	100	YP_010054511.1	8.9
ORF60	–	35,959	35,768	63	Hypothetical protein	Klebsiella virus KpV2811	100	1e-36	100	YP_010054462.1	7.8
ORF61	–	36,258	36,043	71	Hypothetical protein	Klebsiella phage YX3973	100	2e-44	100	YP_010054394.1	8.2
ORF62	+	36,355	36,555	66	Hypothetical protein	*Klebsiella pneumoniae*	100	2e-40	100	WP_142483236.1	8.0
ORF63	+	36,555	37,025	156	Hypothetical protein	Klebsiella phage vB_KpnS_ZX4	100	4e-108	98.72	YP_010054507.1	17.6
ORF64	+	37,025	37,249	74	Hypothetical protein	*Siphoviridae* sp. cthqG28	100	3e-45	97.30	DAD75123.1	8.0
ORF65	+	37,246	37,374	42	Hypothetical protein	*Siphoviridae* sp. cthqG28	100	5e-22	100	DAD75124.1	4.8
ORF66	–	37,540	37,421	39	Hypothetical protein	*Siphoviridae* sp. cthqG28	100	4e-20	97.44	DAD75125.1	4.7
ORF67	–	37,904	37,524	126	Hypothetical protein	Klebsiella phage vB_KpnS_ZX4	100	2e-84	99.21	YP_010054505.1	14.2
ORF68	–	38,124	37,891	77	Hypothetical protein	Vibrio phage pYD38-A	100	2e-46	94.81	YP_008126191.1	8.7
ORF69	–	38,555	38,121	144	TPA: MAG TPA: Lysozyme	*Siphoviridae* sp. cthqG28	100	2e-103	100	DAD75107.1	15.7
ORF70	+	38,654	38,944	96	Hypothetical protein	Klebsiella phage YX3973	100	1e-60	100	YP_010054387.1	9.9
ORF71	+	38,944	39,075	43	Hypothetical protein	Klebsiella phage vB_KpnS_ZX4	100	5e-23	100	YP_010054501.1	5.0
ORF72	+	39,072	39,920	282	Hypothetical protein	*Siphoviridae* sp.	99	2e-139	73.85	DAO12191.1	31.4
ORF73	–	40,948	40,004	314	Hypothetical protein	Klebsiella phage vB_KpnS_ZX4	99	0.0	99.04	YP_010054498.1	35.5
ORF74	–	42,365	40,929	478	Hypothetical protein	Klebsiella phage vB_KpnS_ZX4	100	0.0	97.72	YP_010054497.1	53.6
ORF75	+	42,672	42,878	68	Hypothetical protein	*Klebsiella pneumoniae*	100	4e-38	98.53	WP_116431175.1	7.3
ORF76	+	42,880	43,251	123	TPA: MAG TPA: MerR family regulatory protein	*Siphoviridae* sp.	100	3e-86	99.19	DAF31849.1	13.8
ORF77	–	43,502	43,323	59	Hypothetical protein	*Siphoviridae* sp.	100	2e-32	98.31	DAF31848.1	6.8
ORF78	–	43,738	43,499	79	Hypothetical protein	Klebsiella phage vB_KpnS_ZX4	100	6e-50	100	YP_010054495.1	9.2
ORF79	+	43,829	44,119	96	Hypothetical protein	Klebsiella phage YX3973	100	1e-62	100	YP_010054377.1	10.6
ORF80	–	45,564	44,140	474	TPA: MAG TPA: large terminase	*Siphoviridae* sp.	100	0.0	100	DAZ46203.1	54.2
ORF81	–	46,069	45,557	170	Homing endonuclease	Klebsiella phage YX3973	100	1e-122	100	YP_010054375.1	19.3

#### Replication-, transcription-, regulation-, and packaging-related genes of phage BUCT541

The annotations of the BUCT541 genome sequence indicate that 13 ORFs had functions associated with the replication, translation, and regulation of BUCT541. ORF12, ORF13, and ORF15 are annotated as primase helicase, DEAD/DEAH box helicase, and exodeoxyribonuclease 8, respectively. DEAD-box helicases are a large family of conserved RNA-binding proteins that belong to the broader group of cellular DExD/H helicases. Cellular RNA helicases, including DEAD/DEAH box helicases, have been shown to play roles in recognizing exogenous genes and regulating viral infections (Taschuk and Cherry, 2020). Exodeoxyribonuclease is involved in the RecE recombination pathway by catalyzing the degradation of double-stranded DNA progressively in the 5′ to 3′ direction, releasing 5′-phosphomononucleotides (Murphy, 2012). ORF16 is annotated as an ERF superfamily protein. ERF superfamily proteins have effective recombinase activity and play important roles in genetic recombination engineering (Ricaurte et al., 2018). ORF17 and ORF81 are both annotated as homing endonucleases, which are site-specific endonucleases that initiate homing, a non-reciprocal transfer of its own gene into a new allele that lacks this gene (Wilson and Edgell, 2009). However, the phage T4 endonuclease SegD, which is similar to group I intron endonucleases, did not initiate homing of its own gene or genetic recombination between phages in its site inserted into the rII locus (Sokolov et al., 2018). ORF18 is annotated as a single-strand binding protein. Single-stranded binding proteins bind with high affinity and in a cooperative manner to single-stranded DNA but do not bind well to double-stranded DNA. After binding to single-stranded DNA, these proteins destabilize helical duplexes, thereby allowing DNA polymerases to access their substrate more easily. Single-stranded binding proteins play an important role in the replication of DNA. ORF33, ORF34, and ORF35 are annotated as tRNA-Ser-GCT, tRNA-Arg-TCT, and tRNA-Met-CAT, respectively, implying that large amounts of serine, arginine, and methionine may be required during the lifecycle of BUCT541. ORF46, ORF57, and ORF80 are annotated as putative C-specific methylase, transcription factor, and large terminase, respectively. The terminase large subunit acts as an ATP-driven molecular motor, which is necessary for viral DNA translocation into empty capsids, and as an endonuclease that cuts the viral genome to initiate and end packaging reactions (Leffers and Rao, 2000; Rao and Mitchell, 2001). The phylogenetic tree analysis based on the terminase large subunits constructed by the Neighbor-Joining Algorithm (NJ) method showed that the BUCT541 terminase large subunit shared the highest identity with the terminase large subunits of a *Siphoviridae* sp. virus (GenBank: DAZ46203.1) (Figure 5B).

#### Host cell lysis- and structure-related genes of phage BUCT541

ORF69 is annotated as a lysozyme in the BUCT541 genome. The PSI-BLAST (Position-Specific Iterated BLAST) alignment of the BUCT541 lysozyme amino acid sequence (GenBank: UAW06880.1) with the lysozyme amino acid sequences of closely related viruses showed that the BUCT541 lysozyme sequence shared high coverage (100%) and identity (98.61%) with the *Klebsiella* virus KpV2811 lysozyme sequence (GenBank: YP_010054471.1). ORF19 and ORF31 are annotated as putative tail fiber protein and major tail protein, respectively. Alignment of the BUCT541 tail fiber protein sequence (GenBank: UAW06833.1) with the tail fiber protein sequences of closely related viruses showed that the BUCT541 tail fiber protein shared high cover (100%; *E*-value 0.0) and identity (95.96%) with the *Klebsiella* phage YX3973 tail fiber protein sequence (GenBank: YP_010054359.1). ORF39, ORF52, and ORF53 are annotated as minor capsid protein, coat protein, and capsid stabilizing protein, respectively. The main function of capsid proteins is to protect the stability of DNA (Plano et al., 2021). Because capsid protein sequences are highly conserved, they have been used as a basis for studies into the evolutionary history of phages (Buttimer et al., 2018; Shi et al., 2020). The phylogenetic tree analysis based on the capsid protein constructed by the NJ method showed that the BUCT541 capsid protein had the closest relationship with the *Siphoviridae* sp. minor capsid protein (GenBank: DAE85897.1) (Figure 5C).

### Evaluation of the efficacy of phage BUCT541 against *K. pneumoniae* S-2007 *in vitro*

**Figure 6:**
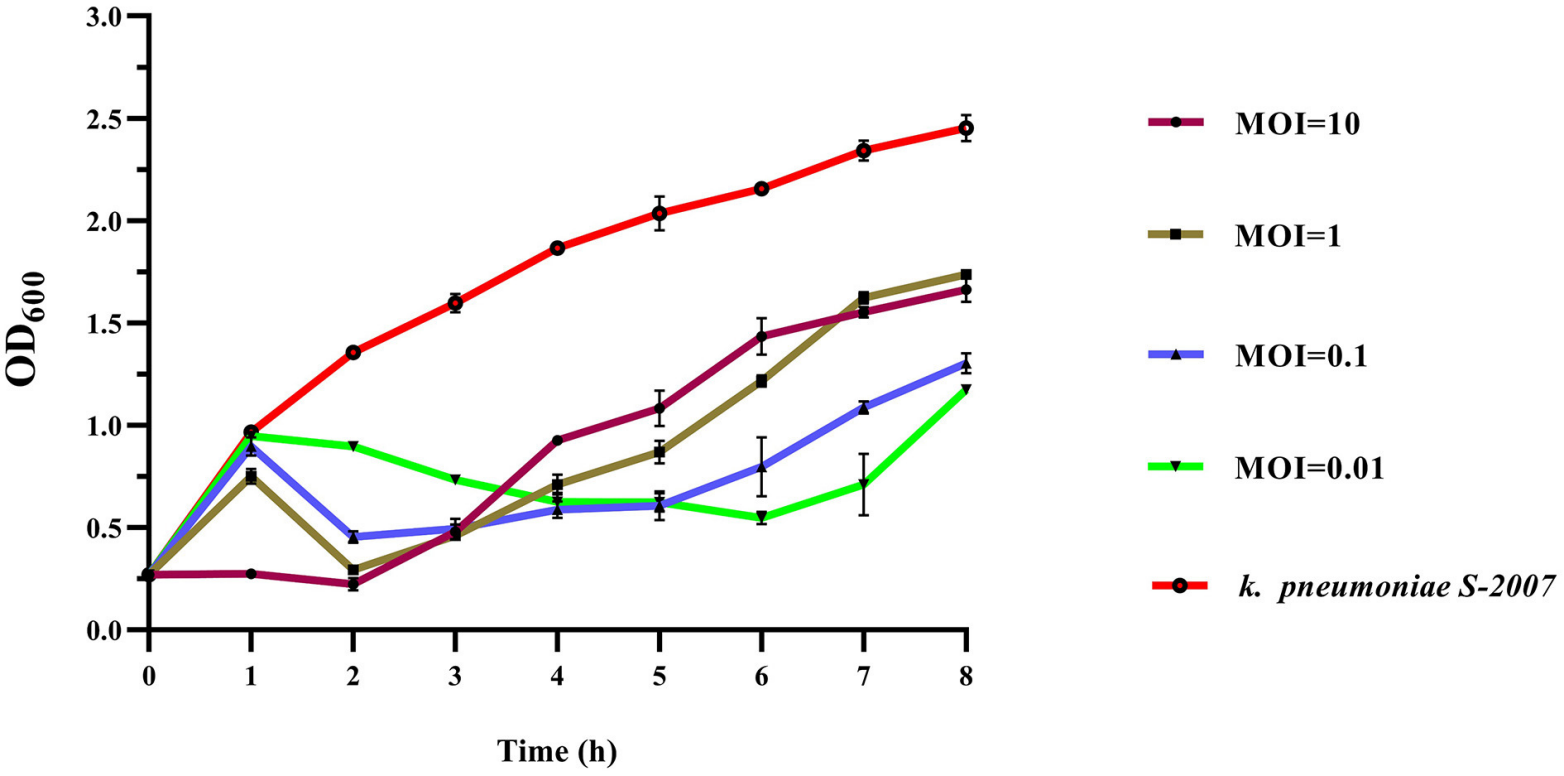
Growth curves of *K. pneumoniae* S-2007 infected with BUCT541 at different MOIs.

During the first hour, the concentration of *K. pneumoniae* S-2007 of the cultures with different MOIs (1, 0.1, 0.01) increased and the increase was basically the same with the control group. However, it decreased sharply at 1 to 2 h. It is noteworthy that with MOI = 10, the concentration of *K. pneumoniae* S-2007 of the cultures was basically unchanged during the first 2 h. After 2 h, the concentration of *K. pneumoniae* S-2007 of the cultures in the different MOIs groups increased again, but the rate of increase was much lower than that of the control group (Figure 6).

### Evaluation of the efficacy of phage BUCT541 to treat *K. pneumoniae* S-2007 *in vivo*

**Figure 7:**
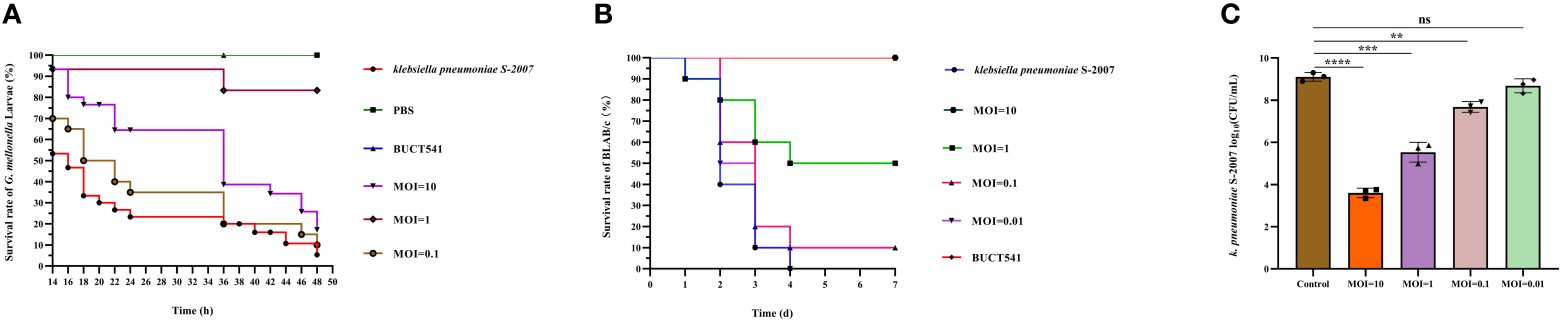
Evaluation of the effect of phage BUCT541 against *K. pneumoniae* S-2007 *in vivo*. **(A)** Survival curves of *G. mellonella* larvae after treatment with phage BUCT541 at different MOIs or PBS. **(B)** Survival curves of mice treated with phage BUCT541 at different MOIs or PBS. **(C)** The amount of *K. pneumoniae* S-2007 in the lungs of mice after 30 h treatment with different MOIs phage BUC541. Data are shown as the mean ± SD, ****P < 0.0001, ***P < 0.001, and **P < 0.01, indicate a significant difference compared to the control group.

**Figure 8:**
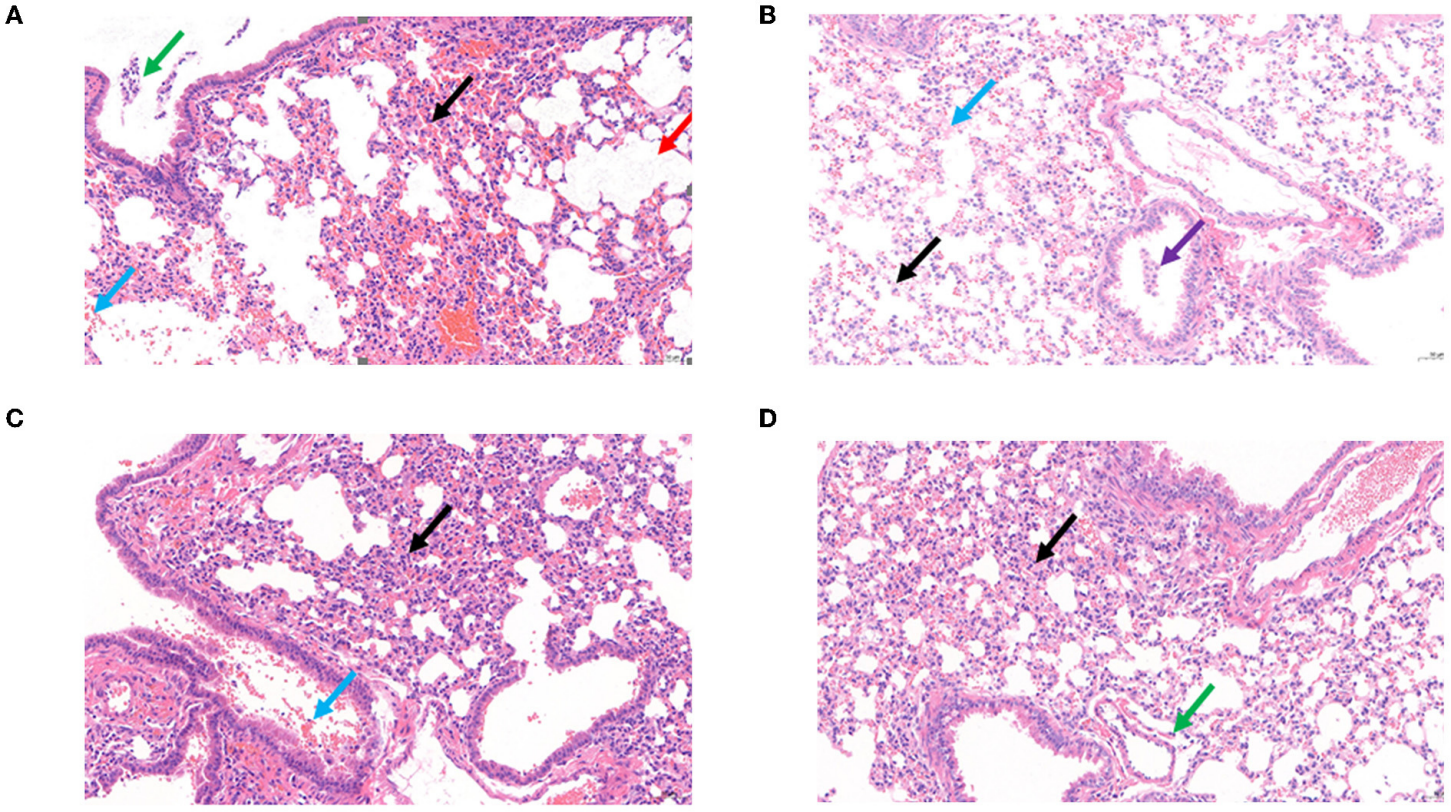
Histopathological examination of the mouse lungs. **(A)** Control: The BALB/c mice only infected *K. pneumoniae* S-2007. **(B)** Negative: The BALB/c mice without any treatment. **(C)** The BALB/c mice infected phage BUCT541(2 × 10^7^ PFU/each). **(D)** Treatment BALB/c mice with MOI = 10 of phage BUCT541. (Black arrow: with a small amount of inflammatory cell infiltration; Blue arrow: multifocal hemorrhage is visible in the tissue; Purple arrow: multiple perivascular edema with a small amount of lymphocytic infiltration; Red arrow: multiple alveolar dilatation is visible in the tissue; Green arrow: a small number of lymphocytes are visible in a few bronchi).

Survival rates of *K. pneumoniae* S-2007-infected *G. mellonella* larvae were 17%, 83.33%, and 10% within 48 h when the MOIs of BUCT541 were 10, 1, and 0.1, respectively (Figure 7A). Almost all of the larvae in the positive control group died within 48 h, and all the larvae in the negative group survived. The survival rates of the BALB/c mice were 100% and 50% within 7 days when the MOIs of BUCT541 were 10 and 1, respectively (Figure 7B). All the mice in the positive control group died within 4 days, and all the mice in the negative group survived. After 30 h treatment with phage BUCT541, the *K. pneumoniae* S-2007 amount in the lungs of positive control mice was as high as 10^9^ CFU/mL, while the amount of S-2007 was lower than 10^4^ CFU/mL when mice were treated with high titers (MOI = 10) of phage BUCT541. The results showed that phage BUCT541 significantly inhibited the growth of *K. pneumoniae* S-2007 in the lungs of mice (Figure 7C). And the histopathologic examination showed that such high titers of BUCT541 significantly reduced the lesions in the mouse lungs and did not affect the normal life activities of the mice (Figures 8A–D). We also noted that infection with BUCT541 alone sometimes caused a mild inflammatory response in the mouse lungs, but this side effect did not pose a threat to the normal activity of the mice (Figure 8C).

## Discussion

Here, we characterized phage BUCT541 and showed that it could lyse MDR-KP K1-ST23, which belonged to the clinically prevalent hvKp. In recent years, the epidemic hvKp K1-ST23 is a big threat as the causative agent in multiple diseases, especially in pyogenic liver abscesses (Chung et al., 2012; Harada et al., 2019). By acquiring mobile elements that carry resistance determinants, hvKp strains have become increasingly resistant to antimicrobial agents (Russo and Marr, 2019). Moreover, the emergence of CR-hvKP caused a higher mortality rate for its characteristic of high virulence and carbapenem resistance, which has further exacerbated the dilemma of antibiotic therapy, triggering the need for alternative therapies (Lan et al., 2021). Phages are considered to be one of the most promising alternative drugs to replace or supplement antibiotic therapy. Therefore, phage BUCT541 with the ability to lyse hvKp K1-ST23 will be a promising treatment option for curing hvKp infection.

BUCT541 formed clearly visible plaques with large diameters on a bacterial lawn of *K. pneumoniae* S-2007. BUCT541 belongs to the *Siphoviridae* family (Ackermann, 2009), as was indicated by electron microscopic and bioinformatics analysis. Interestingly, the electron micrographs showed that the tail terminal of BUCT541 has a special convex structure. Because the phage tail structure is associated with phage adsorption to hosts (Ackermann, 2009), we speculated that the convex structure may be related to the rapid adsorption and high burst of phage BUCT541, and also may be associated with the phage depolymerase. Phage depolymerases are generally located above the tail fiber and tail spike proteins, and changes in the structure of the phage tail fibronectin have been shown to significantly affect the adsorption rate of the phage (Drulis-Kawa et al., 2015; Roach and Donovan, 2015). It has been shown that changes in the structure of the phage tail fibronectin can significantly affect the adsorption rate of the phage (Heller and Braun, 1979). This speculation is consistent with our findings that haloes were distributed around the lysis center of phage BUCT541 and that ORF19 in the BUCT541 genome is annotated as a tail fiber protein. The thermal and pH stability showed that phage BUCT541 is similar to *Klebsiella* phage vB_KpnP_IME279 (Heller and Braun, 1979), which is tolerant to a broad pH range (3–11) and has good thermal stability (4–60°C). Extreme pH conditions affect phage activity by causing irreversible precipitation and coagulation. We found that BUCT541 has good pH and temperature tolerance, which facilitates the storage of BUCT541 and makes it a potential biocontrol agent.

The biological characteristics and safety of phages for phage therapy can be predicted by bioinformatics analysis. The phylogenetic tree based on the complete genomes of BUCT541 and related phages showed that BUCT541 shared the closest evolutionary relationship with the *K. pneumoniae* phage vB_KpnS_ZX4 (GenBank: NC_054654.1), and also had a close evolutionary relationship with *Vibrio* phage pYD38-A (GenBank: JF974312.1) and *Aeromonas* phage pIS4-A (GenBank: NC_042037.1). These results demonstrate the genetic diversity of BUCT541 and provide further information for exploring the genomic evolution of phages (Hatfull and Hendrix, 2011; Reyes and Vives, 2020). ORF 69 of phage BUCT541 was annotated as lysozyme which has a conserved structure and function (Baase et al., 2010). Phage lysozymes have been developed for the detection and prevention of diseases in humans and agriculture (Young, 2014). Notably, ORF33, ORF34, and ORF35 in the BUCT541 genome are annotated as tRNA-Ser-GCT, tRNA-Arg-TCT, and tRNA-Met-CAT, respectively, which is unusual because tRNAs are not commonly found in the genomes of other *K. pneumoniae* phages. We speculate that the replication of BUCT541 may require large amounts of serine (Ser), arginine (Arg), and methionine (Met), which are not efficiently transported by the host, and that BUCT541 evolved the tRNA genes to accelerate the synthesis of Ser, Arg, and Met in the host. Notably, no virulence or drug-resistant genes were annotated in the complete genome of phage BUCT541, suggesting that it may be safe to use BUCT541 as a therapeutic or biocontrol agent; However, the large number of hypothetical proteins requires further investigation.

Phages have natural, safe, and effective strategies to prevent and control multidrug-resistant bacteria, including against the ESKAPE pathogens *Enterococcus faecium, Staphylococcus aureus, K. pneumoniae, A. baumannii, Pseudomonas aeruginosa*, and *Enterobacter* spp. that exhibit multidrug resistance and virulence. In this study, we have assessed the effect of phage BUCT541 against *K. pneumoniae* S-2007 *in vitro* and *in vivo*. *In vitro*, the number of *K. pneumoniae* S-2007 showed a trend of growth followed by decline and finally growth again, which is an arms race phenomenon between phage and host bacteria (Hampton et al., 2020). However, compared with the control group (without phage BUCT541), phage BUCT541 of different MOIs could significantly inhibit the growth rate of *K. pneumoniae* S-2007, especially at MOI = 10. It is possible that phage BUCT541 could be used as a biocontrol agent to contain the spread of *K. pneumoniae in vitro*. *In vivo*, the efficacy of phage BUCT541 against *K. pneumoniae* S-2007 was assessed in the *G. mellonella* and BALB/c mice. *G. mellonella* larvae have been widely used as a model because they are cheap and pose few ethical problems compared with other models (Insua et al., 2013; Wei et al., 2017). We found that the lower doses of BUCT541 phage (MOI = 1) had better therapeutic efficacy than the higher doses (MOI = 10) in *G. mellonella*. This finding is not entirely consistent with previous results that showed that the higher doses of phage led to higher survival rates of *G. mellonella* larvae (Wintachai et al., 2020). We suppose that the lysis of a large number of *K. pneumoniae* may have produced toxic substances such as endotoxin that caused the death of the *G. mellonella* larvae in our study (Luong et al., 2020; Wintachai et al., 2020). This seems to imply that higher phage titers may not always be more effective and the flexibility of phage titers should be considered in phage therapy. To further investigate the potential of phage BUCT541 for clinical application, we assessed the therapeutic effects of phage BUCT541 in BALB/c mice and the ability of BUCT541 to resist *K. pneumoniae* S-2007 in the lungs of mice. The high titer (2 × 10^7^ PFU/each, MOI = 10) of phage BUCT541 completely protects mice infected with *K. pneumoniae* S-2007, and this was quite different from the efficacy of treating *G. mellonella* larvae with high titer phage BUCT541. The different immune systems of the larvae and BLAB/c mice may explain this different outcome. After 30 h of treatment with phage BUCT541 (2 × 10^7^ PFU/each, MOI = 10), *K. pneumoniae* S-2007 in the lungs of mice was below 10^4^ CFU/mL, while *K. pneumoniae* S-2007 in the positive control group reached 10^9^ CFU/mL. It was shown that most of *K. pneumoniae* S-2007 could be cleared by BUCT541 in the mice lungs after 30 h treatment with phage BUCT541. The histopathologic examination of the mouse lungs also showed that phage BUCT541 effectively alleviated lesions caused by *K. pneumoniae* S-2007, and although BUCT541 caused a slight inflammatory response in the BALB/c mice, it did not affect the healthy life activity of the mice. These results suggest that phage BUCT541 can potentially be used as an alternative therapy for drug-resistant *K. pneumoniae* infection.

In conclusion, we characterized phage BUCT541 as a lytic phage against K1-ST23 *K. pneumoniae*. The bioinformatics analysis results and evaluation of therapeutic efficacy in *G. mellonella* larvae and BALB/c mice showed that phage BUCT541 has potential clinical applications in the treatment of *K. pneumoniae* infections.

## Data availability statement

The datasets presented in this study can be found in online repositories. The names of the repository/repositories and accession number(s) can be found in the article/Supplementary material.

## Ethics statement

The animal study was reviewed and approved by Ethics Review Committee of the Seventh Medical Center of the PLA General Hospital.

## Author contributions

MP: resources, data curation, writing—original draft, and investigation. YL: resources, data curation, and investigation. PH and WL: resources and data curation. MP, YL, PH, WL, RG, FQ, XA, LS, and YT: data curation, investigation, and validation. ZC and SZ: supervision and writing—review and editing. HF: conceptualization and supervision. All authors contributed to the article and approved the submitted version.

## Funding

This research was supported by Funds for First-class Discipline Construction (Nos. XK1805 and XK1803-06), National Key Research and Development Program of China (Nos. 2018YFA0903000, 2020YFC2005405, 2020YFA0712100, 2020YFC0840805, 19SWAQ06, and 20SWAQK22), Inner Mongolia Key Research and Development Program (No. 2019ZD006), NSFC-MFST project (China-Mongolia) (No. 31961143024), and Fundamental Research Funds for Central Universities (Nos. BUCTRC201917 and BUCTZY2022).

## Conflict of interest

The authors declare that the research was conducted in the absence of any commercial or financial relationships that could be construed as a potential conflict of interest.

## Publisher's note

All claims expressed in this article are solely those of the authors and do not necessarily represent those of their affiliated organizations, or those of the publisher, the editors and the reviewers. Any product that may be evaluated in this article, or claim that may be made by its manufacturer, is not guaranteed or endorsed by the publisher.
