# Supplementary material for: Genomic characterization of a new phage BUCT541 against Klebsiella pneumoniae K1-ST23 and efficacy assessment in mouse and Galleria mellonella larvae
Source: Front Microbiol. 2022 Sep 16;13:950737. doi: 10.3389/fmicb.2022.950737 (PMC9523250; doi:10.3389/fmicb.2022.950737)
Supplement: Supplementary file 1 [file Table_1.DOCX]

**Table S1.** **primers information**

| **Gene name** | **Sequence** |
| --- | --- |
| **Primers of PCR** | |
| ***rpoB*** | **F: Vic3oF:** GTTTTCCCAGTCACGACGTTGTAGGCGAAATGGCWGAGAACCA  **R: Vic2oR:** TTGTGAGCGGATAACAATTTCGAGTCTTCGAAGTTGTAACC |
| ***gapA*** | **F:** **gapA173oF:** GTTTTCCCAGTCACGACGTTGTATGAAATATGACTCCACTCACGG **R: gapA181oR:** TTGTGAGCGGATAACAATTTCCTTCAGAAGCGGCTTTGATGGCTT |
| ***mdh*** | **F:** **mdh130oF:** GTTTTCCCAGTCACGACGTTGTA CCCAACTCGCTTCAGGTTCAG  **R: mdh867oR:** TTGTGAGCGGATAACAATTTCCCGTTTTTCCCCAGCAGCAG |
| ***pgi*** | **F:** **pgi1FoF:** GTTTTCCCAGTCACGACGTTGTAGAGAAAAACCTGCCTGTACTGCTGGC  **R:** **pgi1RoR:** TTGTGAGCGGATAACAATTTCCGCGCCACGCTTTATAGCGGTTAAT |
| ***phoE*** | **F:** **phoE604.1oF:** GTTTTCCCAGTCACGACGTTGTAACCTACCGCAACACCGACTTCTTCGG  **R: phoE604.2oR:** TTGTGAGCGGATAACAATTTCTGATCAGAACTGGTAGGTGAT |
| ***infB*** | **F: infB1FoF:** GTTTTCCCAGTCACGACGTTGTACTCGCTGCTGGACTATATTCG  **R: infB1RoR:** TTGTGAGCGGATAACAATTTC CGCTTTCAGCTCAAGAACTTC |
| ***tonB*** | **F:** **tonB1FoF:** GTTTTCCCAGTCACGACGTTGTACTTTATACCTCGGTACATCAGGTT  **R:** **tonB2RoR:** TTGTGAGCGGATAACAATTTCATTCGCCGGCTGRGCRGAGAG |
| **Universal sequencing** | **F:** GTTTTCCCAGTCACGACGTTGTA  **R:** TTGTGAGCGGATAACAATTTC |
| ***wzi*** | **F:** **GTGCCGCGAGCGCTTTCTATCTTGGTATTCC**  **R: GAGAGCCACTGGTTCCAGAATTACCGC** |
